# How do different bimaxillary orthognathic surgeries affect facial soft tissues?

**DOI:** 10.1590/1807-3107bor-2026.vol40.072

**Published:** 2026-09-14

**Authors:** Cinthia Oliveira LISBOA, Sergio Luiz MOTA-JÚNIOR, José Thiers CARNEIRO JUNIOR, Eduardo Luis de Sousa CRUZ, Lucia Helena Soares CEVIDANES, Eduardo Franzotti SANT’ANNA, Antonio Carlos de Oliveira RUELLAS, Claudia Trindade MATTOS

**Affiliations:** (a) Universidade Federal Fluminense - UFF, School of Dentistry, Department of Orthodontics, Niterói, RJ, Brazil; (b) Universidade Federal do Rio de Janeiro - UFRJ, School of Dentistry, Department of Orthodontics and Pediatric Dentistry, Rio de Janeiro, RJ, Brazil; (c) Universidade Federal do Pará - UFPA, School of Dentistry, Belém, PA, Brazil; (d) University of North Carolina, Department of Orthodontics, Chapel Hill, NC, USA

**Keywords:** Cone-Beam Computed Tomography, Orthognathic Surgery, Imaging, Three-Dimensional

## Abstract

This study evaluated three-dimensional (3D) soft tissue changes and their correlations with hard tissue movements after combined bimaxillary orthognathic surgery. A retrospective analysis was performed using cone-beam computed tomography scans obtained from 22 adults before surgery (T1) and at least 6 months after surgery (T2). Participants were divided into two groups according to the surgical procedure performed: mandibular setback combined with maxillary advancement (MdSet + MxAdv; n = 10) or mandibular advancement combined with maxillary impaction (MdAdv + MxImp; n = 12). Three-dimensional displacements of hard and soft tissue landmarks were assessed using ITK-SNAP and 3D Slicer software. Pearson’s and Spearman’s correlation coefficients were used to identify associations between hard and soft tissue changes. Significant correlations between anteroposterior hard and soft tissue movements were observed in the subnasal region in the MdSet + MxAdv group (% = 75/70; r = 0.768/0.875) and in the upper lip (% = 83/87/71; r = 0.689/0.760/0.812) and lower lip (% = 54; r = 0.755) regions in the MdAdv + MxImp group. Soft tissue responses in the lower lip and chin regions were significantly correlated with underlying hard tissue movements in both groups; however, the MdAdv + MxImp group demonstrated greater soft tissue response magnitudes (% = 123/136; r = 0.975/0.982) than the MdSet + MxAdv group (% = 75/70; *r* = 0.810/0.727). Significant vertical corr*e*lations in the MdAdv + MxImp group were identified at point A′ (% = 86; r = 0.851) and mandibular landmarks (% = 67/109/108; = 0.855/0.981/0.996), whereas the MdSet + MxAdv group showed significant correlations at the lower lip and Pog′ point (% = 137/73; r = 0.785/0.921). Both groups demonstrated stronger correlations for mandible-related soft tissues in the anteroposterior and vertical directions. These quantified soft-to-hard tissue ratios may improve the prediction of facial soft tissue changes and contribute to more accurate planning of combined bimaxillary orthognathic procedures.

## Introduction

Severe skeletal discrepancies associated with dentofacial deformities are commonly treated with orthognathic surgery. Population-based and institutional studies conducted in different regions have shown that combined bimaxillary procedures account for approximately 39%–65% of all orthognathic surgeries.^1-3^ These procedures are frequently preferred because they permit greater skeletal correction and may provide superior esthetic outcomes.^4^ Among the most commonly performed bimaxillary procedures, mandibular setback combined with maxillary advancement is typically indicated for skeletal Class III malocclusion, whereas mandibular advancement combined with maxillary impaction is often used to treat skeletal Class II malocclusion associated with mandibular deficiency and vertical maxillary excess.

Orthognathic surgery aims not only to correct malocclusion and improve function, but also to enhance facial esthetics, self-esteem, and quality of life.^4-6^ Accurate prediction of soft tissue changes following surgery is therefore highly relevant for patients, surgeons, and orthodontists because these procedures may substantially alter facial proportions and modify the appearance of the nose, lips, and facial contour.^7-10^

Soft tissues generally adapt to underlying skeletal movements; however, the magnitude of soft tissue response varies according to the facial region and type of surgical movement. Previous studies have shown that, following mandibular advancement or setback surgery, the lower lip exhibits a less pronounced response than the chin region.^6^ In addition, Le Fort I osteotomies may significantly affect the middle third of the face, leading to changes in nasal morphology, including alar base width, columellar position, and nasal tip projection.^11-13^ The relationship between hard and soft tissue displacement in the midface is highly variable and may change over time because of factors such as muscular remodeling and residual postoperative edema.^14,15^

For many years, orthognathic surgical outcomes were evaluated using conventional two-dimensional (2D) cephalometric radiographs.^16,17^ However, 2D methods have important limitations in the assessment of facial soft tissue changes because they cannot adequately represent the complexity of three-dimensional facial structures.^18^ In contrast, three-dimensional (3D) imaging modalities—including laser scanners,^10,19-21^ stereophotogrammetry,^22^ digital 3D photogrammetry,^23^ medical computed tomography, and cone-beam computed tomography (CBCT)^6,15,19^—allow more comprehensive and accurate evaluation of facial soft tissue changes after orthognathic surgery.^24^

Among these modalities, CBCT enables superimposition of stable craniofacial structures based on voxel registration, thereby facilitating the assessment of skeletal and soft tissue changes after combined orthodontic-surgical treatment.^24^ Although several studies have used this methodology to evaluate the effects of bimaxillary surgery on facial soft tissues,^24-31^ the variability of soft tissue response according to the type and direction of skeletal movement remains poorly understood.

A previously published systematic review based on 2D cephalometric analyses demonstrated a strong positive correlation between hard and soft tissue changes after mandibular setback surgery.^26^ Another systematic review investigating 3D changes after mandibular advancement or setback surgery reported strong correlations between skeletal and soft tissue displacements in the chin region and moderate correlations in the lower lip/lower incisor region.^27^ Nevertheless, evidence comparing 3D soft tissue responses among different types of combined bimaxillary orthognathic surgeries remains limited.

Therefore, this study aimed to evaluate the correlations and ratios between soft and hard tissue changes following two types of bimaxillary orthognathic surgery: mandibular setback combined with maxillary advancement surgery and mandibular advancement combined with maxillary impaction surgery. The null hypothesis was that soft and hard tissue responses would not differ according to the surgical movement performed.

## Methods

After approval was obtained from the Research Ethics Committee of Universidade Federal Fluminense (CAAE 12130019.5.0000.5243), CBCT scans were retrospectively collected from the Department of Orthodontics at Universidade Federal Fluminense, Universidade Federal do Rio de Janeiro, and a private dental practice. The sample comprised presurgical CBCT scans (T1) and postoperative scans obtained at least 6 months after surgery (T2) from 22 adults (13 women and nine men) aged 18–35 yr who underwent bimaxillary orthognathic surgery. The surgical procedures included mandibular setback combined with maxillary advancement (MdSet + MxAdv) or mandibular advancement combined with maxillary impaction (MdAdv + MxImp).

Eligible participants were adults who had completed bimaxillary orthognathic surgery for skeletal Class II or Class III discrepancies. Pretreatment records were required to demonstrate a minimum overjet of 5 mm, either negative in Class III patients or positive in Class II patients. Patients with cleft lip and palate, craniofacial anomalies, deformities secondary to trauma or degenerative diseases, or CBCT scans with incomplete visualization of facial soft tissues were excluded.

Sample size calculation was based on the correlation coefficient reported by Almeida et al.^6^ (r = 0.86) for the relationship between soft and hard tissue displacement. Assuming 90% statistical power and a significance level of α = 0.05, calculations performed using the website http://estatistica.bauru.usp.br/calculoamostral indicated that a minimum of 10 participants per group was required. The final sample, therefore, consisted of two groups according to surgical procedure: MdSet + MxAdv (*n* = 10) and MdAdv + MxImp (n = 12). A total of 44 CBCT scans were analyzed.

Cone-beam computed tomography images were acquired using an iCat-3D scanner with a 24-cm field of view. DICOM files were converted to “gipl.gz” format using the open-source software ITK-SNAP (version 3.4.0; www.itksnap.org). Voxel size was standardized to 0.4 × 0.4 × 0.4 mm using 3D Slicer software (version 4.10.2; www.slicer.org).

All scans were analyzed using ITK-SNAP and 3D Slicer. Both programs are open-access, user-friendly software packages with extensive tutorials, supporting reproducibility of the methodology. The workflow, adapted from previously published studies,^27-32^ included the following steps:

- Head orientation at T1: Volumetric models were generated, and T1 head orientation was performed by aligning the Frankfurt horizontal and midsagittal planes with the 3D Slicer coordinate system using the “Volume Rendering” and “Transforms” tools (Figure 1).
  **Figure 1:**
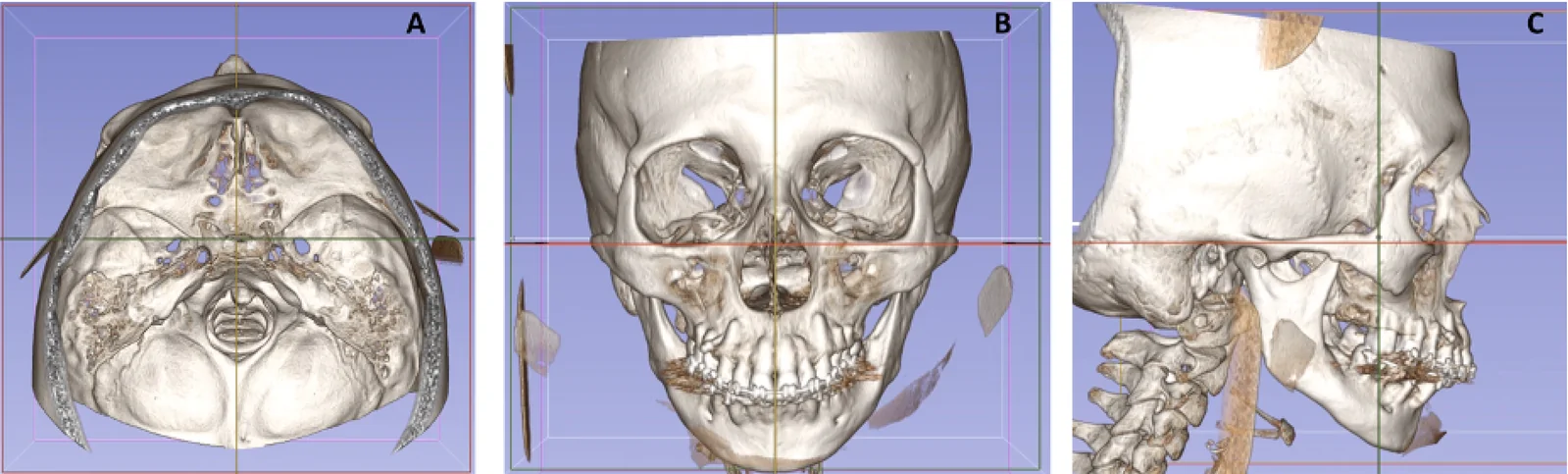
Illustration of head orientation. A, axial view (top); B, frontal view; and C, lateral view.
- Approximation of T1 and T2 scans and skull base segmentation: The T2 scan was initially approximated to the oriented T1 scan using the skull base as a reference. Skull base structures were then segmented in both scans using the “Transforms” tool in 3D Slicer.
- Voxel-based registration: T1 and T2 scans and segmentations were superimposed using the “CMF Registration” tool^30^ in 3D Slicer.
- Landmark identification: The landmarks described in

**Figure 2:**
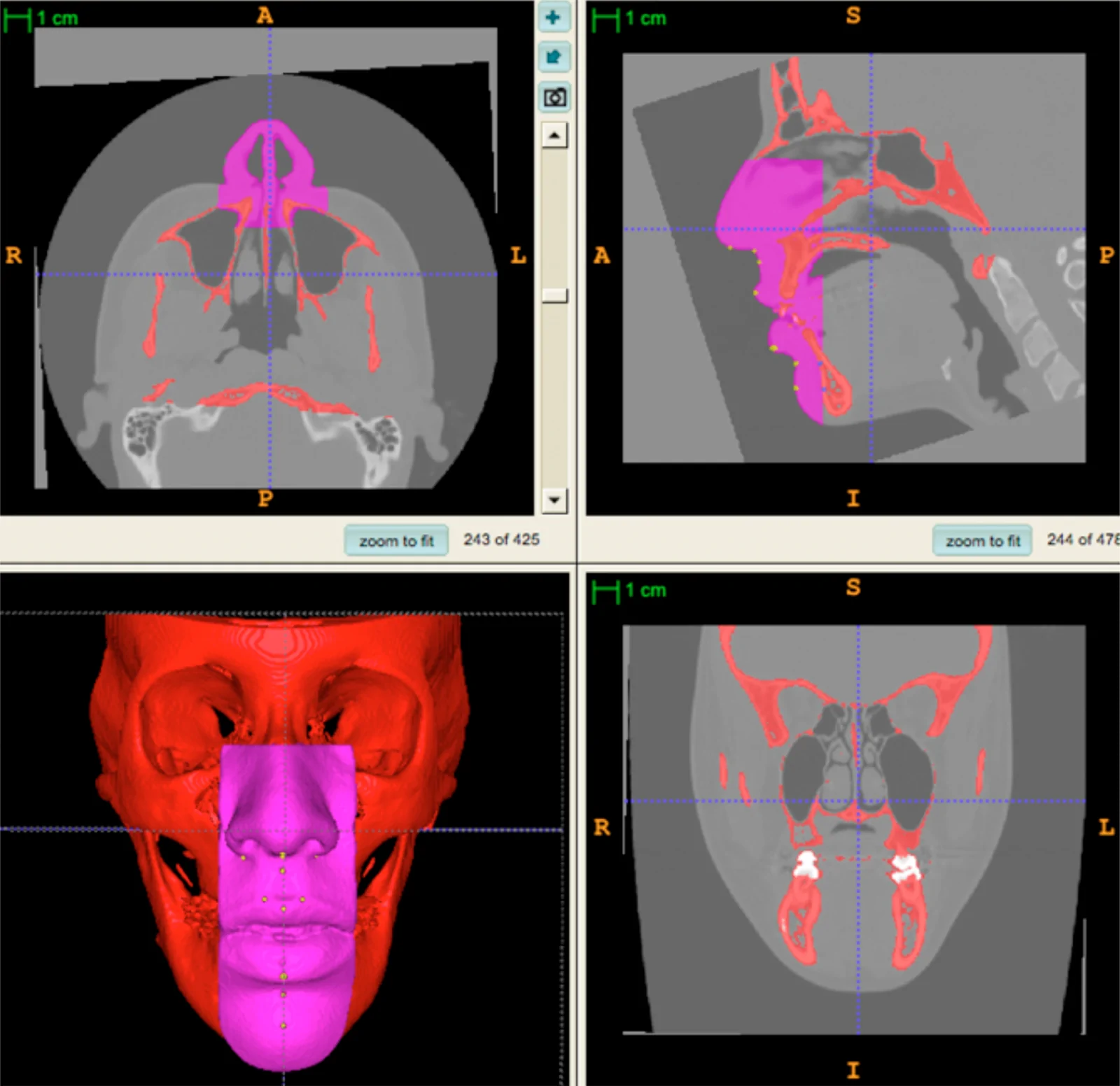
Visualization of facial soft tissue segmentation (pink) and landmark identification. The illustration includes axial, sagittal, and coronal views as well as a 3D model demonstrating the comprehensive approach used for soft tissue landmark placement.

Table 1 were identified on both scans using axial, sagittal, coronal, and 3D views. Soft tissue segmentation was performed to facilitate landmark identification using the “Paintbrush” and “Snake ROI” tools in ITK-SNAP (Figure 2).

**Table 1:** Definition of landmarks used in lateral, axial and coronal visualization

Variables	Definition		
	Lateral view	Axial view	Coronal view
Hard tissue landmarks			
A point (A)	Most posterior point on the curve of the maxilla between the anterior nasal spine and supradent Lt. Al	Middle-anterior-most point on the tip of the premaxilla	Middle point in the anteroposterior slice determined by the lateral and axial views
Upper Incisal Edge (Ui)	Middle point of the incisal edge of the upper central incisor	Middle point of the mesiodistal and buccolingual width	Middle point of the mesiodistal width
Lower Incisal Edge (Li)	Middle point of the incisal edge of the lower central incisor	Middle point of the mesiodistal and buccolingual width	Middle point of the mesiodistal width
B point (B)	Most posterior point of the anterior surface of the mandibular symphysis	Middle-anterior-most point on the anterior contour	Middle point in the anteroposterior slice determined by the lateral and axial views
Pogonion (Pog)	Most anterior point on the contour of the bony chin	Middle-anterior-most point on the anterior contour of the bony chin	Middle point in the anteroposterior slice determined by the lateral and axial views
Soft tissue landmarks			
Columela (Cm)	Anterior-inferior most point on the nose	Middle point of the inferior region of the nose	Middle point in the anteroposterior slice determined by the lateral and axial views
Subnasale (Sn)	Point between the nose and point A’	Middle point of the nose base	Middle point in the anteroposterior slice determined by the lateral and axial views
Soft A point (A’)	Most posterior point between Sn e LS in soft tissue	Middle point between Sn and LS in the soft tissue	Middle point in the anteroposterior slice determined by the lateral and axial views
Left alar base (Lt. Al)	Most lateral point in the curved base line of the alar on the left side. indicating the facial insertion of the nasal wing base	Most lateral point in the curved base line of the alar on the left side. indicating the facial insertion of the nasal wing base	Most lateral point in the curved base line of the alar on the left side. indicating the facial insertion of the nasal wing base
Right alar base (Rt. Al)	Most lateral point in the curved base line of the alar on the right side. indicating facial insertion of the nasal wing base	Most lateral point in the curved base line of the alar on the right side. indicating facial insertion of the nasal wing base	Most lateral point in the curved base line of the alar on the right side. indicating facial insertion of the nasal wing base
Upper Lip (UL)	Most prominent point of the upper lip	Middle-anterior point in the upper lip	Middle point in the upper lip
Left cupid’s bow (Lt. Cup)	_	_	Most superior point of the vermilion border of the left cupid bow
Right cupid’s bow (Rt. Cup)	_	_	Most superior point of the vermilion border of the right cupid bow
Lower lip (LL)	Most prominent point of the lower lip	Middle-anterior-most point on the lower lip	Middle point in the lower lip
Soft B point (B’)	Most posterior point of soft tissue of the mandibular symphysis	Middle-anterior-most point on the soft tissue of the mandibular symphysis	Middle point in the anteroposterior slice determined by the lateral and axial views
Soft Pogonion (Pog’)	Most anterior point on the contour of the soft tissue chin	Middle-anterior-most point on the anterior contour of the soft tissue chin	Middle point in the anteroposterior slice determined by the lateral and axial views

- Conversion to 3D surface models: Skull segmentations and landmarks were converted into 3D surface models using the “Model Maker” tool in 3D Slicer.
- Linear measurements: Three-dimensional landmark displacement in mm between T1 and T2 was calculated using the Q3DC tool^32^ in 3D Slicer. Measurements included Euclidean distance as well as displacement along the anteroposterior (y-axis), vertical (z-axis), and transverse (x-axis) planes.
- Angular measurements: Nasolabial (Cm-Sn-UL) and mentolabial (Li-B′-Pog′) angles were measured at both timepoints using the Q3DC tool in 3D Slicer.

All measurements were performed by a single examiner (COL) who was previously trained by an experienced researcher (ACOR). Both evaluators were blinded to patient identity and demographic characteristics. However, blinding of postoperative scans regarding surgical group allocation was not feasible because the surgical changes were clearly identifiable in the images.

To assess reproducibility, scans from eight participants were randomly selected and reanalyzed after a 2-week interval. Intraexaminer reliability for landmark placement and measurements was evaluated using the intraclass correlation coefficient (ICC). Reliability was classified as excellent (> 0.90), good (0.75–0.90), moderate (0.50–0.75), or poor (< 0.50).^33^

Statistical analyses were performed using Jamovi software (version 1.6.3; https://www.jamovi.org/). Data normality was assessed using the Shapiro–Wilk test. The chi-square test was used to compare sex distribution between groups. The one-sample *t*-test or Wilcoxon signed-rank test was used to evaluate whether intragroup changes between timepoints differed significantly from zero. Intergroup comparisons between surgical procedures were performed using the independent *t*-test or Mann–Whitney test, as appropriate. Changes in angular measurements between preoperative and postoperative timepoints within each group were assessed using the paired *t*-test.

Pearson’s or Spearman’s correlation coefficients were calculated to determine associations between hard and soft tissue changes. Correlation strength was classified as very strong (> 0.90), strong (0.70–0.90), moderate (0.50–0.70), or weak (< 0.50).^34^ Ratios between hard and soft tissue displacements were calculated to express the percentage of soft tissue response relative to underlying hard tissue movement. Linear regression analysis was performed for statistically significant correlations, with soft tissue landmark displacement considered the dependent variable.

## Results

Table 2 presents the demographic characteristics and distribution of the sample. According to the chi-square test, no statistically significant differences were observed in sex distribution between the groups (p = 0.665).

**Table 2:** Demographic characteristics and distribution of the sample according to the surgical groups.

Variable	MdSet + MxAdv	MdAdv + MxImp	Total
**n = 10**	**n = 12**	**n = 22**
Age range (yr)	18–35	20–35	18–35
Sex, *n* (%)			
Female	5 (50)	8 (66.7)	13 (59.1)
Male	5 (50)	4 (33.3)	9 (40.9)

MdSet + MxAdv, mandibular setback with maxillary advancement; MdAdv + MxImp, mandibular advancement with maxillary impaction.

The ICC demonstrated good-to-excellent reliability for all repeated measurements. For soft tissue landmarks, repeatability ranged from 0.793 (Sn) to 0.969 (UL). For hard tissue landmarks, ICC values ranged from 0.815 (point A) to 0.902 (Pog). The ICC values were 0.866 for the maxillary incisor (Ui) and 0.933 for the mandibular incisor (Li).

Table 3 summarizes the descriptive statistics for anteroposterior (AP), vertical, transverse, and three-dimensional (3D) changes in both surgical groups, as well as the corresponding intra- and intergroup comparisons. Statistically significant AP changes from T1 to T2 (p < 0.05) were observed for several maxillary and mandibular soft tissue landmarks (Cm, Sn, A′, Lt. Cup, Rt. Cup, LL, B′, and Pog′) in both surgical groups. Significant intergroup differences were identified for LL (p = 0.014), B′ (p = 0.014), and Pog′ (p = 0.014). Significant vertical changes from T1 to T2 were observed for Cm, Sn, and LL in the MdSet + MxAdv group and for all maxillary and mandibular soft tissue landmarks in the MdAdv + MxImp group, except B′ and Pog′. A significant intergroup difference was found only for LL (p = 0.016). Although both surgical approaches primarily aimed to produce AP and vertical skeletal changes, transverse displacements were also evaluated as part of the comprehensive 3D analysis. No statistically significant transverse differences were observed in either group, indicating overall transverse stability. Three-dimensional displacements from T1 to T2 were statistically significant for all evaluated landmarks in both groups. However, the only significant intergroup difference was identified for Pog′, indicating that both surgical protocols produced changes of similar magnitude, except for this region, which showed a greater magnitude of change in the MdAdv + MxImp group (p = 0.048).

**Table 3:** Mean values and standard deviations (SD) in mm, medians of changes in the anteroposterior, vertical, and transverse directions, and 3D changes between preoperative (T1) and postoperative (T2) scans for both surgical groups.

Variables	Anteroposterior changes					Vertical changes					Transverse changes					3D changes				
	MdSet+MxAdv		MdAdv+MxImp		Intergroup	MdSet+MxAdv		MdAdv+MxImp		Intergroup	MdSet+MxAdv		MdAdv+MxImp		Intergroup	MdSet+MxAdv		MdAdv+MxImp		Intergroup
	Mean (SD)	Median	Mean (SD)	Median	p-value	Mean (SD)	Median	Mean (SD)	Median	p-value	Mean (SD)	Median	Mean (SD)	Median	p-value	Mean (SD)	Median	Mean (SD)	Median	p-value
Hard tissue landmarks																				
A	4.03 (2.59)*	3.89	1.36 (1.21)*	0.84	0.005*	0.29 (1.33)	0.32	3.90 (4.03)*	2.28	0.037*	-0.20 (0.86)	-0.14	-0.37 (1.21)	-0.21	0.705	4.38 (2.47)*	4.40	4.88 (3.89)*	3.10	0.106
Ui	4.07 (2.76)*	3.11	2.18 (3.61)*	3.61	0.234	-0.23 (2.23)	-0.14	4.93 (4.11)*	3.49	0.014*	-0.51 (1.73)	-0.31	-0.26 (1.76)	-0.34	0.740	5.09 (2.38)*	4.89	6.69 (4.00)*	6.39	0.645
Li	-2.92 (2.56)*	-3.26	7.25 (4.19)*	7.55	0.014*	2.22 (2.23)*	2.40	3.57 (5.47)*	2.49	0.635	-1.01 (2.45)	-0.45	-1.00 (2.67)	-0.01	0.993	5.34 (2.11)*	4.99	8.29 (4.48)*	7.76	0.379
B	-3.57 (3.60)*	-2.78	5.85 (2.35)*	5.33	0.014*	2.99 (3.47)*	4.46	2.59 (5.08)	1.19	0.833	-1.24 (1.61)	-0.64	-0.59 (2.72)	-0.21	0.520	6.37 (2.89)*	5.29	8.96 (5.31)*	7.84	0.586
Pog	-2.47 (2.84) *	-2.28	7.25 (4.19)*	7.55	0.014*	2.55 (3.70)	3.32	0.87 (5.55)	1.01	0.617	-1.17 (1.57)	-0.48	-0.43 (2.70)	-0.08	0.453	5.46 (2.58)*	6.00	12.00 (7.14)*	11.50	0.168
Soft tissue landmarks																				
Cm	1.80 (1.99)*	1.85	1.87 (2.22)*	1.18	0.933	1.80 (2.07)*	2.46	2.47 (2.69)*	1.88	0.649	-0.10 (0.93)	0.13	-0.05 (0.72)	-0.08	0.878	3.38 (1.85)*	3.52	3.56 (3.05)*	2.41	0.927
Sn	2.82 (1.98)*	2.43	1.89 (1.86)*	1.44	0.289	1.58 (1.02)*	1.84	2.60 (2.88)*	1.70	0.537	-0.21 (0.62)	-0.17	-0.34 (0.69)	-0.22	0.652	3.51 (1.82)*	3.19	3.56 (3.13)*	2.61	0.967
A’	3.02 (3.07)*	3.02	1.67 (1.17)*	1.48	0.229	1.28 (3.01)	0.96	3.36 (3.77)*	2.35	0.348	-1.19 (3.37)	0.04	-0.60 (1.42)	-0.26	0.589	5.05 (3.91)*	4.21	4.50 (3.34)*	3.27	0.985
Lt.Al	4.04 (1.66)*	4.37	1.09 (1.83)	0.52	0.002*	1.05 (1.38)	1.16	2.40 (4.40)*	1.62	0.579	3.34 (6.66)	1.39	-0.45 (2.08)	-0.25	0.076	6.69 (5.58)*	5.37	4.33 (3.79)*	3.99	0.677
Rt.Al	4.29 (1.94)*	4.39	1.14 (1.98)	0.49	0.004*	0.40 (1.36)	2.30	2.49 (4.15)*	1.47	0.393	-0.74 (4.51)	-0.94	-0.88 (2.17)	-0.41	0.920	5.90 (3.46)*	5.25	4.45 (3.66)*	4.04	0.706
UL	3.64 (2.22)*	3.99	1.54 (2.74)	2.23	0.104	0.09 (2.21)	0.23	2.39 (4.25)*	1.67	0.445	-0.10 (0.71)	0.10	-0.37 (1.25)	-0.21	0.552	4.33 (1.95)*	4.19	4.47 (3.40)*	4.04	0.985
Lt.Cup	3.23 (2.10)*	3.07	1.89 (2.54)*	2.59	0.227	1.09 (2.20)	0.35	3.19 (3.85)*	2.34	0.379	-0.45 (1.35)	-0.82	-0.41 (2.26)	-0.64	0.953	4.31 (1.90)*	3.97	5.04 (3.72)*	3.64	0.926
Rt.Cup	3.59 (2.10)*	3.60	1.81 (2.57)*	2.24	0.138	0.96 (2.43)	0.25	3.38 (3.83)*	2.71	0.400	-0.09 (1.16)	-0.38	-0.30 (1.29)	-0.21	0.702	4.60 (1.86)*	4.69	4.86 (3.64)*	4.00	0.965
LL	-1.94 (1.83)*	-1.97	3.94 (2.99)*	4.11	0.014*	-3.47 (4.27)*	-2.89	3.85 (5.10)*	2.36	0.016*	-1.12 (1.66)	-0.50	-0.61 (1.35)	-0.45	0.438	5.60 (2.90)*	4.76	6.75 (4.54)*	6.13	0.887
B’	-2.66 (3.14)*	-2.56	7.22 (4.76)*	6.47	0.014*	1.86 (4.46)	2.68	2.84 (4.80)	1.57	0.718	-1.05 (1.55)	-0.35	-0.47 (2.56)	0.01	0.541	5.86 (2.64)*	5.41	8.84 (5.75)*	6.81	0.588
Pog’	-1.73 (2.49)*	-2.08	9.93 (6.96)*	10.10	0.014*	1.80 (3.28)	2.83	0.94 (5.49)	0.83	0.713	-1.11 (1.65)	-0.38	-0.57 (2.71)	0.11	0.589	5.09 (2.37)*	5.20	12.80 (6.76)*	11.80	0.048*

Significant intragroup differences are indicated. *p*-values for intergroup displacement differences are derived from the independent *t*-test or Mann–Whitney test, with the Benjamini–Hochberg correction applied. Directions and sign conventions for changes from T1 to T2: Anteroposterior changes: (+) anterior movements, (-) posterior movements. Vertical changes: (+) superior (upward) movements, (-) inferior (downward) movements. Transverse changes: (+) movements toward the left and (-) movements toward the right. * Significant difference (p < 0.05).

Table 4 presents the nasolabial and mentolabial angle measurements obtained at the preoperative (T1) and postoperative (T2) timepoints for both groups, including mean differences and corresponding p-values. No statistically significant changes in the nasolabial angle were observed after surgery in either group (MdSet + MxAdv: mean difference = 1.72°, p = 0.588; MdAdv + MxImp: mean difference = −6.19°, p = 0.075). In contrast, statistically significant reductions in the mentolabial angle were observed in both groups after surgery. The MdSet + MxAdv group demonstrated a mean decrease of 11.7° (p = 0.005), whereas the MdAdv + MxImp group showed a mean decrease of 10.1° (p = 0.001), indicating a consistent postoperative change in the mentolabial region regardless of the surgical protocol.

**Table 4:** Mean values and standard deviations (SD) of angular measurements recorded at the preoperative (T1) and postoperative (T2) timepoints, along with the mean differences observed between these timepoints

Variables	MdSet + MxAdv	MdAdv + MxImp
**Mean (SD)**	**Mean (SD)**
Nasolabial angle (NL)		
T1	102 (17.4)	117 (18.6)
T2	104 (13.8)	111 (13.8)
T2-T1	1.72 (10.7)	-6.19 (10.9)
p-value (T2-T1)	0.588	0.075
Mentolabial angle (Lme)		
T1	157 (11.4)	149 (19.5)
T2	146 (11.8)	139 (18.4)
T2-T1	-11.7 (10.0)	-10.1 (7.03)
p-value (T2-T1)	0.005^*^	0.001^*^

The table also presents *p*-values for the mean differences between T1 and T2 within each group, as obtained from paired *t*-tests. *Significant difference.

**Figure 3:**
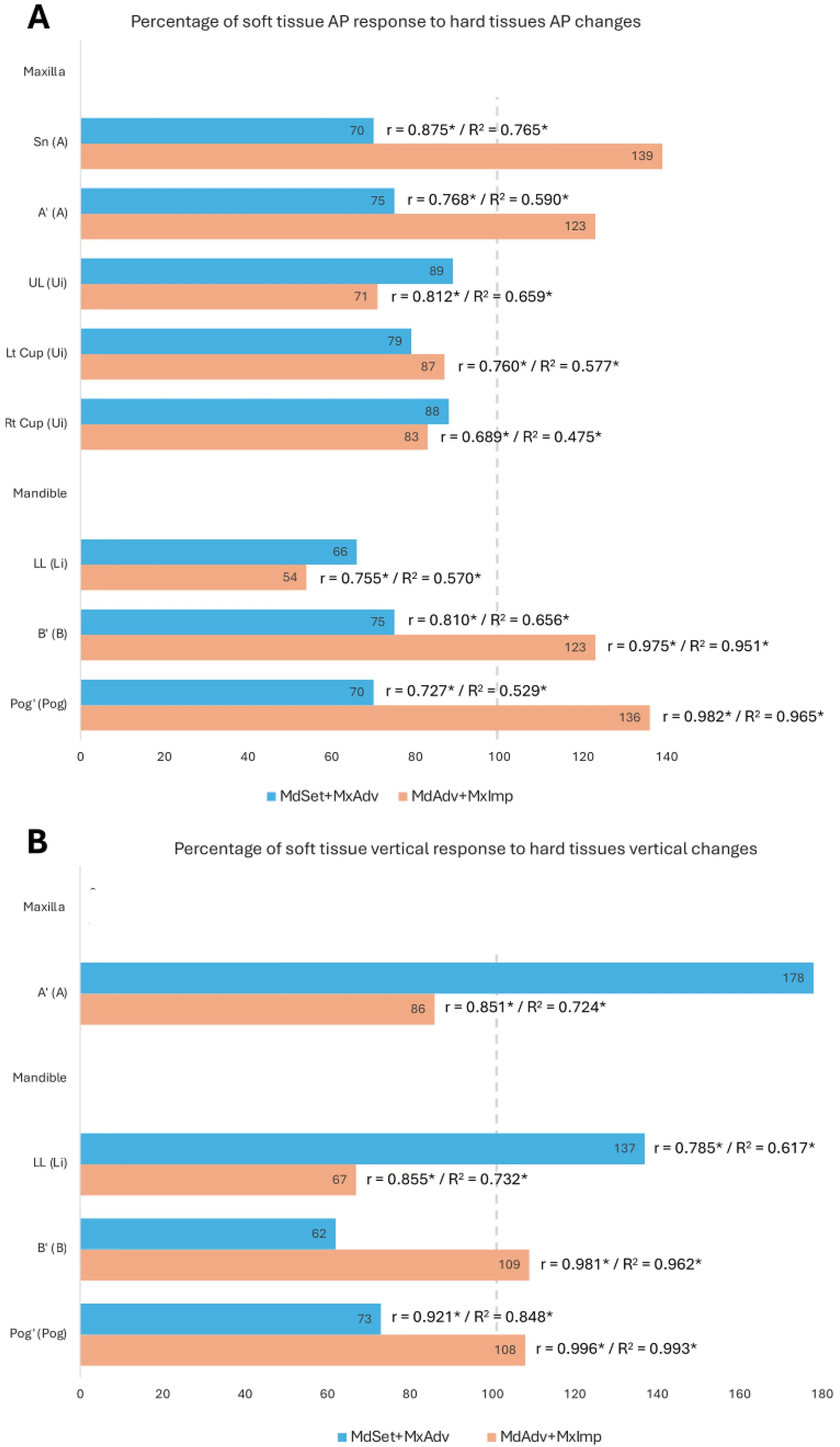
A, Graphs depicting the percentage of anteroposterior (AP) soft tissue response relative to AP hard tissue changes; B, Graphs depicting the percentage of vertical soft tissue response relative to vertical hard tissue changes. Each graph identifies the evaluated soft tissue landmark, with the corresponding hard tissue landmark indicated in parentheses. Hard tissue changes were normalized to 1 (100%) for ratio calculation. Statistically significant correlation (r) and linear regression (*R*2) coefficients are displayed in the graphs. p < 0.05

Figure 3A illustrates the relationship between the percentage of soft tissue response in the AP direction and AP changes in the corresponding hard tissues, including correlation (*r*) and linear regression (*R* ^2^) coefficients when statistically significant. In the subnasal region, soft tissue response rates ranging from 70% to 75% were significantly correlated in the MdSet + MxAdv group. In the MdAdv + MxImp group, upper lip and lower lip soft tissue responses to AP hard tissue changes ranged from 71% to 87% and 54%, respectively, and showed significant correlations. In the lower lip and chin regions, response rates of 70%–75% were significant in the MdSet + MxAdv group, whereas higher response rates of 123%–136% were observed in the MdAdv + MxImp group (Table 5).

**Table 5:** Correlation coefficients (*r*) and *p*-values between hard and soft tissue landmarks in the anteroposterior direction and displacement rate between hard and soft tissues.

Variables	Cm		Sn		A’		Lt. Al		Rt. Al	
	MdSet+ MxAdv	MdAdv+ MxImp	MdSet+ MxAdv	MdAdv+ MxImp	MdSet+ MxAdv	MdAdv+ MxImp	MdSet+ MxAdv	MdAdv+ MxImp	MdSet+ MxAdv	MdAdv+ MxImp
A coef. corr.	0.476	0.493	0.875	0.154	0.768	0.213	0.500	0.156	-0.039	0.043
p-value	0.300	0.163	0.010*	0.695	0.033*	0.697	0.310	0.769	0.915	0.894
ratio	1:0.45	1:1.37	1:0.70	1:1.39	1:0.75	1:1.23	01:01	1:0.80	1:1.06	1:0.84
	UL		Lt. Cup		Rt. Cup					
Ui coef. corr.	0.154	0.812	0.173	0.760	0.331	0.689				
p-value	0.738	0.004*	0.774	0.011*	0.550	0.023*				
ratio	1:0.89	1:0.71	1:0.79	1:0.87	1:0.88	1:0.83				
	LL									
Li coef. corr.	0.319	0.755								
p-value	0.507	0.011*								
ratio	1:0.66	1:0.54								
	B’									
B coef. corr.	0.810	0.975								
p-value	0.027*	0.005*								
ratio	1:0.75	1:1.23								
	Pog’									
Pog coef. corr.	0.727	0.982								
p-value	0.047*	0.005*								
ratio	1:0.70	1:1.36								

*Significant difference.

Figure 3B presents the percentage of vertical soft tissue responses relative to vertical hard tissue changes, together with correlation (*r*) and linear regression (*R* ^2^) coefficients when statistically significant. Soft tissue landmarks in the subnasal region demonstrated an 86% response rate that was significantly correlated in the MdAdv + MxImp group. The lower lip showed a response rate of 67% in the MdAdv + MxImp group and 137% in the MdSet + MxAdv group, both significantly correlated with underlying hard tissue changes. For the B′ and Pog′ landmarks, vertical soft tissue responses ranged from 108% to 109% in the MdAdv + MxImp group and from 62% to 73% in the MdSet + MxAdv group (Table 6).

**Table 6:** Correlation coefficients and *p*-values between hard and soft tissue landmarks in the vertical direction and displacement rate between hard and soft tissues.

Variables	Cm		Sn		A’		Lt. Al		Rt. Al	
	MdSet+ MxAdv	MdAdv+ MxImp	MdSet+ MxAdv	MdAdv+ MxImp	MdSet+ MxAdv	MdAdv+ MxImp	MdSet+ MxAdv	MdAdv+ MxImp	MdSet+ MxAdv	MdAdv+ MxImp
A coef. corr.	0.430	0.336	0.624	0.580	0.600	0.851	0.648	0.413	0.467	0.524
p-value	0.300	0.287	0.132	0.095	0.134	0.003*	0.135	0.202	0.279	0.154
ratio	0.16:1	1:0.63	0.18:1	1:0.66	0.22:1	1:0.86	0.27:1	1:0.61	0.72:1	1:0.64
	UL		Lt. Cup		Rt. Cup					
Ui coef. corr.	0.309	0.490	0.081	0.399	0.137	0.399				
p-value	0.470	0.173	0.824	0.276	0.776	0.276				
ratio	1:0.39	1:0.48	0.21:1	1:0.64	0.24:1	1:0.68				
	LL									
Li coef. corr.	0.785	0.685								
p-value	0.038*	0.047*								
ratio	0.63:1	1:0.67								
	B’									
B coef. corr.	0.689	0.944								
p-value	0.099	0.003*								
ratio	1:0.62	0.91:1								
	Pog’									
Pog coef. corr.	0.921	0.993								
p-value	0.010*	0.003*								
ratio	1:0.73	0.92:1								

*Significant difference.

## Discussion

Combined bimaxillary orthognathic surgeries have become increasingly common because they allow greater skeletal correction.^4,24^ Consequently, understanding the effects of these procedures on facial esthetics is clinically important.

In the MdSet + MxAdv group, the middle third of the face, including the upper lip and maxillary incisor regions, shifted anteriorly, corroborating previous 3D imaging studies demonstrating that maxillary advancement is associated with anteroposterior (AP) changes in the nose and upper lip.^13,16^ In contrast, mandibular hard and soft tissues, including the lower lip and mandibular incisors, moved posteriorly. Previous studies using both 2D and 3D imaging have shown that soft tissues generally follow underlying hard tissue movements after mandibular setback and clockwise rotation procedures, although variability in postoperative stability and esthetic outcomes has been reported.^4,35,36^

In the MdAdv + MxImp group, all evaluated landmarks moved upward and forward, with significant changes particularly observed in mandibular landmarks in the AP direction and at point A as well as in the vertical displacement of the incisors. Maxillary impaction surgery, commonly indicated for correction of vertical maxillary excess, may induce counterclockwise mandibular rotation even in the absence of mandibular surgery. However, the combination of mandibular advancement and counterclockwise rotation appears to contribute to more pronounced skeletal and soft tissue changes.^14,37^

The correlation between AP displacement of hard and soft tissues was strong and statistically significant between hard tissue point A and the soft tissue landmarks Sn and A′ only in the MdSet + MxAdv group. Linear regression analysis demonstrated that 76.5% of the variation at Sn and 59% at A′ could be explained by the anterior movement of point A. A systematic review^37^ reported considerable variability in the AP displacement ratio between point A and point Sn after maxillary advancement, ranging from 1:0.06 to 1:0.86. In the present study, the ratio between point A and point Sn displacement was 1:0.70.

In the MdAdv + MxImp group, no significant AP correlation was observed between point A and the corresponding soft tissue landmarks, likely because maxillary impaction procedures typically involve limited AP displacement of point A. Ratios between soft and hard tissue displacements after combined bimaxillary surgery tend to be more variable in the middle third of the face,^14^ especially around the nasal and paranasal regions. This variability may be associated with progressive muscular remodeling and relaxation of the nasolabial musculature rather than surgical relapse.^14,15,37,38^

In the vertical direction, a strong correlation was identified between hard tissue point A and soft tissue point A′ in the MdAdv + MxImp group. Linear regression analysis indicated that 72.4% of the vertical displacement of point A′ was explained by skeletal movement at point A. This finding was expected because superior repositioning of the maxilla was a primary objective in correction of vertical maxillary excess. Conversely, no significant correlations or regression values were observed for vertical displacement in the MdSet + MxAdv group, likely because vertical maxillary changes were not part of the surgical objective.

For the MdAdv + MxImp group, strong AP correlations were identified between maxillary incisor displacement and the UL, Lt. Al, and Rt. Al soft tissue points, whereas vertical correlations were weak. Linear regression analysis demonstrated that 65.9% of upper lip AP movement could be explained by maxillary incisor displacement. In contrast, weak correlations were observed in the MdSet + MxAdv group for both AP and vertical relationships between maxillary incisor movement and the upper lip. This finding agrees with previous research^36^ suggesting that maxillary advancement may have a limited influence on upper lip AP position because the upper lip is affected by several additional factors, including tissue thickness, muscle tone, and individual biologic variability.^39^

The MdAdv + MxImp group also demonstrated a strong AP correlation and a moderate vertical correlation between mandibular incisor and lower lip displacements. Linear regression analysis confirmed these findings, indicating that anterior incisor movement produced approximately 57% corresponding movement in the lower lip. In the MdSet + MxAdv group, however, a strong correlation was observed only for vertical changes. A systematic review^26^ reported a moderate correlation between mandibular incisors and lower lip displacement following mandibular advancement or setback surgery. Another systematic review^14^ found an approximate 50% horizontal displacement ratio between the lower lip and mandibular incisors after combined bimaxillary surgery.

For both surgical groups, strong AP correlations were identified between skeletal and soft tissue mandibular landmarks (B-B′ and Pog-Pog′). Linear regression analysis demonstrated that, following mandibular advancement, soft tissue landmarks B′ and Pog′ closely followed—and in some cases exceeded—the displacement of the underlying skeletal landmarks B and Pog. After mandibular setback surgery, mandible-related soft tissues followed skeletal movement at approximately 70%–75%. In the vertical dimension, the MdSet + MxAdv group demonstrated a strong correlation for Pog-Pog′, whereas the MdAdv + MxImp group exhibited very strong correlations for all mandibular landmarks. Previous studies have shown that mandibular soft tissues generally follow underlying skeletal movements predictably after mandibular advancement or setback surgery, whether performed alone or combined with maxillary procedures,^14^ particularly in the horizontal direction.^40^ Nevertheless, the present results demonstrated a greater magnitude of soft tissue response to mandibular skeletal displacement in the MdAdv + MxImp group.

Vertical analyses of mandibular soft tissue changes are less frequently reported and are generally more complex than AP analyses.^15^ Compared with the midface and lips, mandibular soft tissues appear less affected by factors such as muscular adaptation, facial expression, incisor position, tissue thickness, and soft tissue tonicity.

In the present study, ratios between AP mandibular hard and soft tissue displacements were greater than those observed between mandibular incisors and lower lip movement in both groups. These findings support previous systematic review data indicating that soft tissue pogonion (Pog′) displacement nearly mirrors hard tissue pogonion displacement (1:0.98), whereas the ratio between mandibular incisors and lower lip displacement is approximately 1:0.78.^26^ Facial musculature appears to exert greater influence on lower lip position and behavior than skeletal and dental structures,^41^ regardless of the surgical procedure performed.

Both surgical groups demonstrated comparable magnitudes of 3D soft tissue displacement. Because 3D displacement represents Euclidean distance, this measure reflects movement magnitude without accounting for movement direction or qualitative surgical characteristics. Therefore, the absence of significant intergroup differences suggests that both surgical protocols produced soft tissue changes of similar magnitude despite differences in surgical movement patterns. However, the retrospective design of this study should be considered when interpreting and generalizing the findings.

The severity of the skeletal discrepancy and characteristics of the facial soft tissues should both be considered during treatment planning and selection of the surgical approach. Although orthognathic diagnosis traditionally divides the face into thirds, the potential for global facial changes may sometimes be underestimated. Isolated mandibular surgery may influence the nose and upper lip, whereas Le Fort I osteotomy may also affect the lower lip and chin.^37^ These findings reinforce the concept that the face functions as an integrated unit and that all facial regions should be carefully evaluated during orthognathic surgical planning.^42^

## Conclusions

When comparing soft tissue response to hard tissue repositioning after bimaxillary surgery, this study demonstrated differences in soft tissue response ratios according to the type of surgical movement performed:

- Anteroposterior hard tissue movements were significantly correlated with soft tissue response in the subnasal region in the MdSet + MxAdv group and in the upper and lower lips in the MdAdv + MxImp group. The lower lip and chin regions showed significant correlations in both groups, although the MdAdv + MxImp group demonstrated greater soft tissue response magnitudes.
- Vertical hard tissue movements were significantly correlated with soft tissue response at point A′ and mandibular landmarks in the MdAdv + MxImp group and at the lower lip and Pog′ point in the MdSet + MxAdv group.
- These findings provide clinically relevant information for planning combined bimaxillary orthognathic procedures. Quantified soft-to-hard tissue response ratios may improve the prediction of postoperative facial changes and support virtual surgical planning and patient communication regarding esthetic outcomes. Nevertheless, individual variation in soft tissue characteristics highlights the importance of comprehensive preoperative assessment and realistic patient expectations.

## Data Availability

The datasets generated during and/or analyzed during the current study are available from the corresponding author on reasonable request.
